# Contact Bubble Bilayers with Flush Drainage

**DOI:** 10.1038/srep09110

**Published:** 2015-03-16

**Authors:** Masayuki Iwamoto, Shigetoshi Oiki

**Affiliations:** 1 Department of Molecular Physiology and Biophysics, University of Fukui Faculty of Medical Sciences, Fukui 910-1193, Japan

## Abstract

Planar lipid bilayers have been used to form stable bilayers into which membrane proteins are reconstituted for measurements of their function under an applied membrane potential. Recently, a lipid bilayer membrane is formed by the apposition of two monolayers that line an oil-electrolyte interface. Here, a bilayer membrane system is developed with picoliter bubbles under mechanically and chemically manipulable conditions. A water bubble lined with a phospholipid monolayer is blown from a glass pipette into an oil phase. Two blowing pipettes are manipulated, and bubbles (each with a diameter of ~ 50 μm) are held side by side to form a bilayer, which is termed a contact bubble bilayer. With the electrode implemented in the blowing pipette, currents through the bilayer are readily measured. The intra-bubble pressure is varied with the pressure-controller, leading to various sizes of the bubble and the membrane area. A rapid solution exchange system is developed by introducing additional pressure-driven injection pipettes, and the blowing pipette works as a drain. The solution is exchanged within 20 ms. Also, an asymmetric membrane with different lipid composition of each leaflet is readily formed. Example applications of this versatile method are presented to characterize the function of ion channels.

The lipid bilayer membrane is a self-organized structure, and biological evolution in an aqueous environment led to the formation of bilayer membranes from phospholipids to compartmentalize cellular contents. The fluidity and deformability of the bilayer membrane enables changes in the cellular shape, leading to variations in cell function. Similar self-organized membranes are easily formed in our daily life as soap bubbles in air from a detergent solution, although the layer arrangement of the membrane is reversed^1^,^2^. The principles of the self-assembly of amphipathic molecules are common to both bilayer membranes and soap bubbles, and knowledge from soap bubbles has been applied to the formation of artificial bilayer membranes^1^,^3^,^4^,^5^.

Lipid bilayer membranes are readily formed as planar lipid bilayers (PLBs). PLBs have been formed on a small hole made on hydrophobic supporting material using various techniques^3^,^4^,^5^. In addition, a bilayer was formed on the tip of a glass pipette by “patch-clamping” a preformed bilayer^6^,^7^. This variety of techniques offers opportunities for utilizing a method that is suitable for experimental purposes.

Recently, methods were developed for forming a bilayer membrane by using the interface between an aqueous solution and organic solvents^8^,^9^,^10^,^11^,^12^,^13^,^14^. Monolayers that form at the interface between oil and water phases are positioned face to face, and a bilayer is generated. In the original method, a microfluidic flow system was designed for guiding electrolyte and oil phases^8^. Simpler methods were developed, in which a water droplet was immersed in an oil phase and the monolayers that formed at the interface between the water and oil phases were positioned near each other to form the bilayer, which is named the droplet interface bilayer (DIB)^12^.

These lipid bilayer systems have been exploited to study membrane proteins. Among them, ion channels are the main target of these studies because channels mediate the ion flux across the membrane. Because of ready applicability of the voltage clamp technique, the lipid bilayer system is a highly accurate current measurement system. To study the molecular mechanism of channel function, such as selective ion permeation and gating, it is rational to study channel proteins on artificial membranes, with a composition that can be controlled, rather than on cell membranes, with a composition that varies. Channels have been reconstituted into lipid bilayer membranes, and extensive studies involving single-channel current measurements have been performed^15^,^16^.

Here, we developed a lipid bilayer system to characterize channel function under various manipulable conditions, such as in a variety of lipid compositions and with the application of stable membrane potentials. The system exploits the flexible oil-electrolyte interface used in the DIB and manipulable glass pipettes used in the patch-clamp technique. Two water bubbles are blown into an oil phase from the tips of two pipettes (30 μm in diameter each), and each bubble is lined with phospholipid monolayers at the oil-water interface. These bubbles are maneuvered such that the monolayers are positioned side by side to form a bilayer. The size of the bubble is readily changed using the pressure control from the pipette. We call this system the contact bubble bilayer (CBB) system. In the CBB, the bubble size is small (picoliter), but the bubble interior is connected to the bulk pipette solution, which allowed development of a rapid solution exchange system. The CBB method combines the easy physical manipulability (pressure and tension control) of patch clamping and the chemical manipulability (lipid composition changes in each leaflet level) of the bilayer method. Some applications of the CBB method are presented.

## Results and discussion

**Figure 1:**
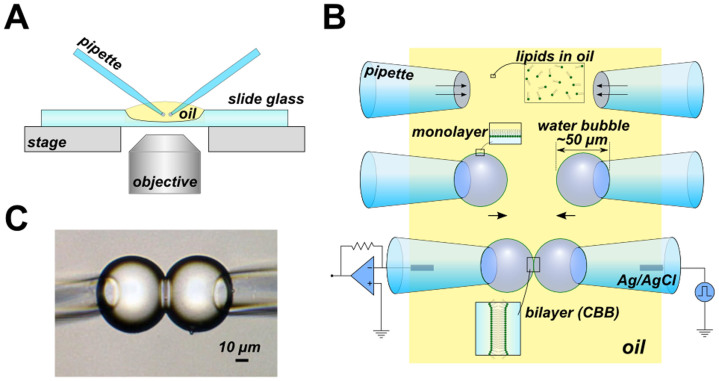
The simplest CBB method. (A) Overview of the CBB system on the microscope. All observations and measurements were performed on an inverted microscope (IX70, Olympus). (B) Schematic illustration of the procedure for the CBB formation. The phospholipids are contained in the oil phase (e.g., 20 mg/mL azolectin in hexadecane). Two glass pipettes filled with electrolyte solutions are dipped into the lipid-containing oil. The lipid in the oil phase is spontaneously transferred to the oil-electrolyte interface, and a monolayer is formed there. The electrolyte bubbles surround the tips of a pair of glass pipettes upon the application of pipette pressure (middle). Two drops contact each other, forming a CBB (lower). (C) Photograph of the CBB. The bilayer is observed from the tangential direction. The central line delineates the bilayer, which has a diameter of approximately 30 μm.

The procedure for forming the CBB is as follows (Fig. 1). A small amount of oil (hexadecane) is used to fill a concave slide glass, and all the procedures are performed and observed using an inverted microscope. In the simplest maneuver, the phospholipids are dispersed in the oil phase beforehand, and the electrolyte-containing pipettes with a tip diameter of 30 μm are dipped into the oil phase. The phospholipids transfer spontaneously from the bulk oil phase to the oil-electrolyte interface at the tip. As pressure is applied, a water bubble grows at the tip, and the pressure is then fixed. Without the phospholipids, a bubble does not form, similarly to the impossibility of blowing a soap bubble in the air; low surface tension is required^1^,^2^. At the appropriate lipid concentration (e.g., 20 mg/mL azolectin in hexadecane), the size of the bubbles was kept constant by decreasing the pressure applied in the pipettes relative to that at the blowing moment. Two bubbles are placed side by side using motor-driven manipulators, and as the bubbles approach one another, the monolayers are attracted to each other to form a bilayer spontaneously via inter-surface attraction^17^. The size of the bilayer can be controlled by adjusting the distance between the pipettes and the intra-bubble pressure. Additionally, the curvature of the membrane is controlled by varying each bubble size according to the Plateau law^18^,^19^,^20^. The formation of the bubble and the maintenance of its size are controlled by pressure through the pipettes using a pressure control device. The thickness of the bilayer is governed by the organic solvent, and in the case of hexadecane, the specific capacitance was 0.83 μF/cm^2^ (see Supplement Fig. S1), indicating that the formed bilayer was nearly solvent-free^21^.

The phospholipids in the oil-electrolyte monolayer are also served from the aqueous phase. Liposomes are contained in each pipette solution, and they are spontaneously extended as a monolayer at the oil-electrolyte interface of each pipette. Then, the bubbles are blown in the lipid-less oil. Channel molecules are included in the pipette solution as a solubilized form or as proteoliposomes. Solubilized channels are spontaneously transferred to the bilayer. When proteoliposomes are used, the channels are also distributed on the monolayer at the oil-electrolyte interface and are transferred to the bilayer phase once the bilayer is formed^22^. Channel-bearing liposomes are also likely fused to the bilayer.

In the CBB, the small space inside the bubbles is connected to the bulk pipette solution, which functions as the bath. Thus, the CBB, including the interiors of both bubbles and the external pipette solution, is considered a thermodynamically open system. Depletion of the ionic species in bubbles during high flux experiments is not a concern.

The smaller size of the bubble relative to that in the DIB system, and thus the smaller membrane area (< 100 μm^2^), substantially improves the electrical signal-to-noise ratio. In the supplement, the root-mean-square noise was plotted as a function of the membrane area (Fig. S1). The CBB experiments are usually performed for a bubble diameter of 50 μm and a membrane area of 80 μm^2^; for this condition, the noise level (0.4 pA rms at 2 kHz cut-off frequency) is close to the system background noise. Single-channel or macroscopic current recordings with maximal signal-to-noise ratio can be recorded. The CBB is durable; a stable membrane is maintained even under a membrane potential as high as ± 400 mV. Higher membrane potentials occasionally disrupt the bilayer, causing two bubbles to merge; under this condition, the electrode potential of the AgCl can be balanced. The pipette can be repeatedly used, and refreshed bubbles readily form a bilayer.

The attaching and detaching of the monolayers can be repeated by manipulating the bubbles mechanically. In this reversible process, what happens to the membrane-spanning channels? Here, we apply the peptide channel known as polytheonamide B (pTB) from marine sponge^23^,^24^ to monitor the detach–attach process. The pTB channel is a 48-mer peptide, which forms a β^6.3^-helix with 4-Å inner pore diameter and allows permeation of monovalent cations^25^,^26^. When added to an aqueous solution, such as in the right side bubble, the pTB channel is vectorially inserted into the membrane; the hydrophobic N-terminal end serves as the lead, while the hydrophilic C-terminal end remains in the aqueous phase. Thus, the pTB channels are incorporated into the CBB with a uniform orientation, and the macroscopic current exhibits asymmetric current–voltage relationships^25^.

**Figure 2:**
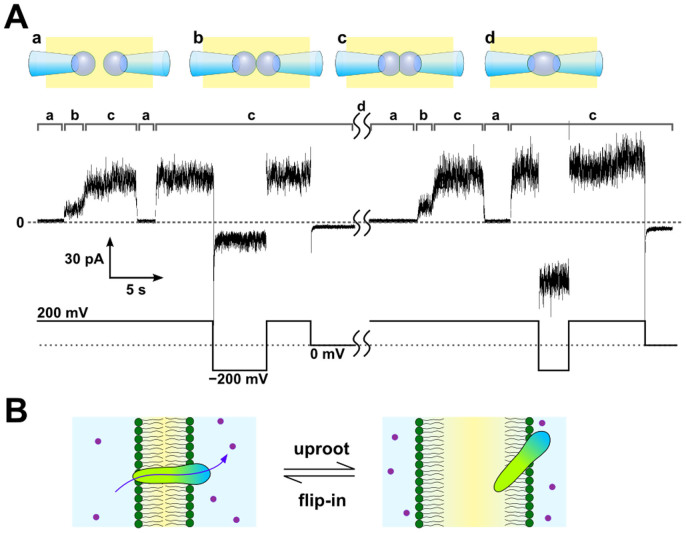
Channel activity of the pTB channel during the attach–detach process of the CBB. (A) Time course of the channel current during the bilayer formation and detachment. Configurations of the bubbles (a–d) are illustrated above (upper panel). The time course of the bubble manipulation (indicated by the alphabet) and the corresponding current traces (middle panel) at the applied membrane potential (lower panel) are shown. pTB was added inside the right bubble (100 pM). Once the bilayer was formed (b), the pTB channels penetrated into the membrane with the same orientation, and the macroscopic current was detected immediately. As the two bubbles were pushed closer to each other (c), the membrane area increased, leading to a gradual increase in the current amplitude. Then, the two bubbles were separated from each other (a), and the current amplitude decreased to the null level. Reformation of CBB restored the macroscopic current of the pTB channel. After reformation, the channel orientation was not reversed, as shown by the asymmetric current amplitudes at ± 200 mV where the rectification factor (*I*_+200mV_/*I*_−200mV_) was 3.33 ± 0.03 (*n* = 4, ± SEM). Once the CBB broke by applying high voltage (e.g., 1 V) between two electrodes (d), the solution inside both bubbles mixed. After mixing, pTB penetrated into reformed CBB from both bubbles, which was confirmed by symmetric current amplitudes at ± 200 mV (right half period, rectification factor was 1.06 ± 0.09, *n* = 6, ± SEM). The aqueous buffer contained 3 M CsCl and 10 mM HEPES, and the pH was adjusted to 7.5. (B) Schematic illustration of the pTB channel during the attach–detach process.

Fig. 2A is a representative macroscopic current trace of the pTB channel under indicated membrane potentials. At the beginning, two bubbles were not attached (a) and only the right side bubble contained the pTB channel. After the attachment of the bubbles (b), the current appeared immediately and then increased gradually as the membrane area increased (c) and the number of membrane-incorporated channels also increased until reaching a steady-state current level. Then, the pipettes were pulled away, and the membrane was detached. During the detaching process, the current gradually decreased because the bilayer area decreased and the membrane-embedded channels were uprooted. When the bubbles were attached again, the current increased immediately and reached the same steady-state current level. The smaller inward current amplitude at − 200 mV compared to 200 mV indicates that the asymmetric current was maintained (Fig. 1A, left half), and thus the incorporated channels maintain the same orientation as before. Once the CBB was disrupted by applying high membrane potential (e.g., 1 V), two bubbles were merged, and pTB diffused homogeneously inside a bubble (d). When a CBB is reformed after the merge, current amplitudes at ± 200 mV became symmetrical (Fig. 1A, right half).

The simplest interpretation of this result is the following (Fig. 2B). Upon detaching, the membrane-embedded pTB channels were withdrawn exclusively to the membrane leaflet of the right side, where they remained on the monolayer until the bilayer was formed again. If the membrane embedded pTB would be uprooted to the left leaflet, they are incorporated into the reformed bilayer lead by the N-term, giving the opposite orientation. Also, if pTB could be transferred into the oil phase, it is partitioned to both leaflets. Thus, orientations of the channels were mixed, and the macroscopic current amplitudes should not retain the asymmetry at ± 200 mV after the membrane detachment. The present experimental result indicates that the pTB channels had been uprooted to the monolayer of the right bubble exclusively, and they were flipped into the bilayer membrane upon CBB reformation, reproducing the asymmetric channel activity as before. The hydrophilic C-terminus of the pTB channel anchored the channel to the membrane leaflet of the right side and enabled the channel to flip in and out when the bilayer fused and split. The reversible and repeated detachment and attachment of monolayers in the CBB enabled this unprecedented experiment and elucidated the pTB's membrane insertion mechanism.

**Figure 3:**
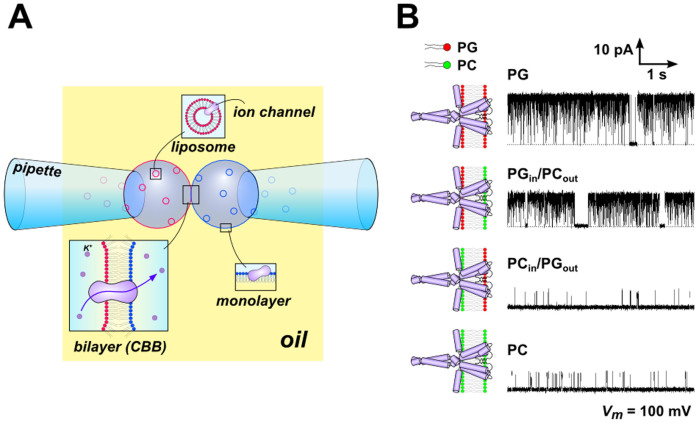
The asymmetric CBB and the KcsA channel activity. (A) Schematic illustration of the formation of the asymmetric CBB. Each bubble, formed in the lipid-less oil (hexadecane), contained different types of KcsA-containing liposomes, in which the lipid composition was either phosphatidylcholine (PC) or phosphatidylglycerol (PG). In this case, the PG_in_/PC_out_ asymmetric membrane was formed. pH of the electrolyte solution in two bubbles were also set asymmetric, such as pH of the left bubble is acidic and that of the right bubble is neutral. Consequently, only channels oriented with their cytoplasmic side to the left bubble elicit channels' electric signal. (B) Typical single-channel current traces of the KcsA channel in symmetric and asymmetric membranes at 100 mV. The open probabilities ware 92 ± 2%, 88 ± 4%, 5 ± 2% and 10 ± 3% in the PG, PG_in_/PC_out_, PC_in_/PG_out_ and PC membrane, respectively (*n* = 3, ± SEM). Each aqueous solution contained 2 mg/mL proteoliposome, 200 mM KCl and succinic acid (pH 4.0, the left bubble) or 10 mM HEPES (pH 7.5, the right bubble). The lipid-protein ratio (w/w) of the proteoliposome was 2000.

In the CBB, an asymmetric membrane^4^,^27^ is readily formed with the following method. Liposomes having different lipid compositions are placed in the pipette solutions, and the lipids are transferred to the oil-water interface to form the monolayer. The water bubbles are then blown in the lipid-free hexadecane (Fig. 3A). Apposition of the bubbles leads to the formation of the asymmetric membrane. For reconstitution of the channel proteins in the asymmetric bilayer, the channels were reconstituted into liposomes made of the relevant lipid constituent beforehand, and the channels were distributed on the extended monolayer or fused to the CBB.

Here, we demonstrate the formation of an asymmetric membrane, in which the lipid dependence of the KcsA potassium channel was characterized (Fig. 3). Previously, the effect of phospholipids on the KcsA channel were examined using an asymmetric lipid bilayer formed by the folding method in a planar bilayer^28^, but a similar asymmetric membrane was attained more easily in the CBB. Proteoliposomes consisting of either phosphatidylglycerol (PG) or phosphatidylcholine (PC) were placed in either bubble (Fig. 3B). The inactivation-free mutant of the KcsA channel, E71A, was used for examining the activation gate. The mutant channels were transferred from the proteoliposomes to the CBB, and a single-channel current was recorded. In the symmetric membrane, the open probability (*P*_open_) was 92% in the PG membrane but was only 10% in the PC membrane. The high *P*_open_ was retained when PG was used in the membrane inner leaflet (left bubble) but not when it was used in the outer leaflet (right bubble). Thus, we obtained the consistent results of the KcsA channel in the asymmetric CBB more efficiently^28^.

**Figure 4:**
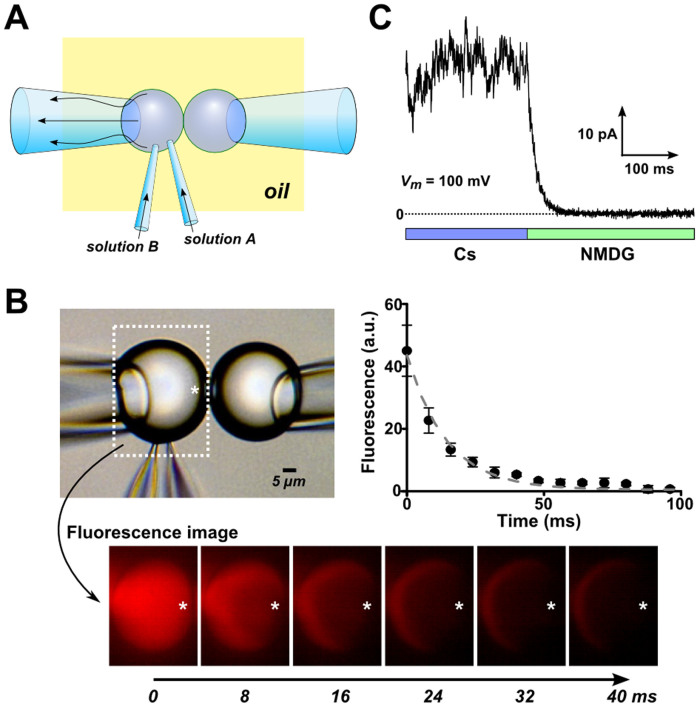
Perfusion of the CBB. (A) Schematic illustration of the solution exchange inside the bubble. Two glass pipettes penetrated a bubble, and different solutions were injected from the two pipettes. The pressure inside the bubble-holding pipette was adjusted to keep the size of the bubble constant. Therefore, the flow from the injection pipettes was drained toward the bubble-holding pipette. (B) Visualization of the perfusion inside the bubble by use of fluorescence probe. The left bubble contained fluorescence probe and non-fluorescence solution started to flow from the injection pipette at time zero. Snapshots of the fluorescence image during perfusion were shown below. Fluorescence intensity inside the bubble at the indicated position (*) was quantified from the snapshots and the time course was presented on the right side. Decay time constant of the fluorescence intensity was 14.4 ± 0.4 ms (*n* = 3, ± SEM). (C) Typical time course of the current through the pTB channel. The pTB channel was incorporated into the CBB, and the macroscopic current was measured in the symmetrical CsCl solution at 100 mV. Then, the solution was changed to the NMDG-Cl solution, and the current decayed as the Cs was washed out with a time constant of 17.7 ± 1.7 ms (*n* = 5, ± SEM).

In the CBB, the bubble size is small (picoliter), but the bubble interior is connected to the bulk solution in the bubble-holding pipette, which allowed development of a rapid solution exchange system. Two fine pipettes (injection pipettes) containing different solutions penetrated one of the bubbles (here the left side bubble; Fig. 4A). One of the perfusion pipettes (pipette A) was filled with a solution of the desired composition, and the other pipette (pipette B) was filled with the same solution as that in the left side bubble. The pressure inside the bubble-holding pipette was adjusted to keep the size of the bubbles constant. The perfusion started when pressure was applied to pipette A. The solution in the bubble was rapidly exchanged for solution A, and the pre-existing solution was drained to the bubble-holding pipette. Subsequently, a pressure injection of the original solution from pipette B immediately washed out solution A and switched to the original composition. Thus, the concentration can be changed in a pulse-like manner, and we call this system the rapid perfusion-and-drainage system.

The speed of the solution exchange was evaluated using the following methods. First, the solute concentration inside the perfusing bubble was visually monitored over time using a fluorescence probe (Qdot) (Fig. 4B). The left bubble contained fluorescence probe and non-fluorescence solution started to flow from the injection pipette at time zero. Fluorescence intensity inside the bubble was quantified from snapshots of the fluorescence images (Fig. 4B, lower panels) and the time course is demonstrated (Fig. 4B, right panel). The washout starts from the right side in the bubble, where the fluorescence intensity was measured, and drained towards the holding pipette. Second, the time course of the solution exchange near the channel was monitored by the conductance changes of the pTB channel (Fig. 4C). The macroscopic pTB current was measured upon the exchange of the solution from the permeating CsCl solution to the non-permeating NMDG-Cl solution. In this method, the changes in the current amplitude should reflect the Cs^+^ concentration at the vicinity of the membrane-embedded channel. Both fluorescence and current results indicate that the time constant of the solution exchange was faster than 20 ms, and this value was extremely faster than the previous report using DIB^29^.

**Figure 5:**
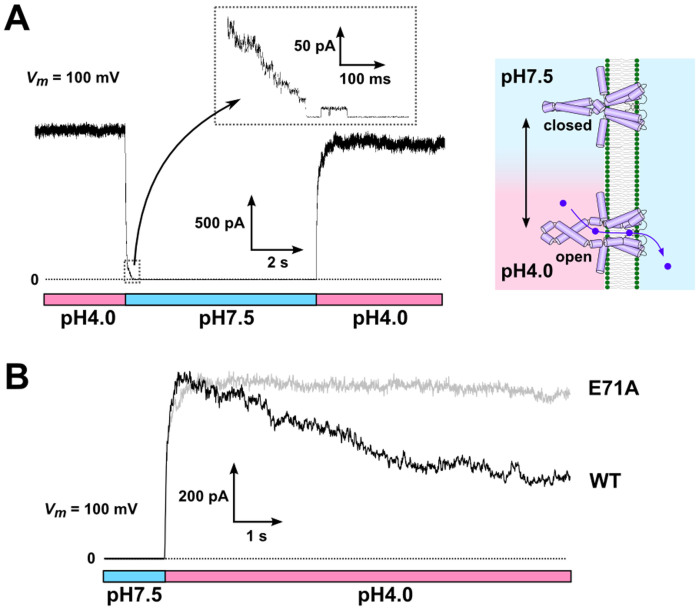
Time course of the pH-dependent gating of the KcsA channel in response to a pH-jump. (A) Time course of the macroscopic current of the E71A mutant of the KcsA channels at 100 mV. The pH of the intracellular side of the channel was changed from 4.0 to 7.5 and then returned to 4.0 as indicated below the current trace. In response to the neutral pH jump, the current decayed rapidly with a time constant of 30 ms. A fraction of the channels remained open after the pH change for more than 200 ms (inset). Sidedness of proton activation of the KcsA channel is illustrated (right panel). (B) Time course of the activation for the WT and E71A mutant channels. The acidic jump elicited the activation of both the WT and E71A channels with a similar time course, but only the WT channel showed a subsequent slow inactivation. The current trace of the WT was ensemble averaged (*n* = 5) and the decay time constant was 3.97 ± 0.02 s (± SEM). The membrane potential was kept at 100 mV during the recordings.

In the last section, we examined the pH-dependent gating of the KcsA channel using the rapid perfusion-and-drainage system. In earlier studies, solution exchanges were performed using the liposome-patch method^30^, and the time course of the pH-dependent activation and inactivation gating of the KcsA potassium channel was evaluated^31^. Here, we applied the non-inactivating mutant of KcsA, E71A^32^, and the time course of the activation and deactivation of the channel gating was examined using the rapid perfusion-and-drainage system under an experimental condition similar to that for the liposome-patch. In the liposome-patch experiment, the mixed lipid azolectin was exclusively used for the liposome. While arbitrary lipid compositions can be used in the CBB, we formed the CBB in similar condition as the liposome-patch experiments. Proteoliposomes with a high protein : lipid weight ratio (1 : 20) were placed in the pipette solution, and the macroscopic KcsA current was recorded under an asymmetric pH condition (the left bubble: 4.0; the right bubble: 7.5) (Fig. 5A). In this configuration, the channels whose cytoplasmic side faced the left side were active and contributed to the current signal. When the pH of the left solution was changed to 7.5, the current attenuated rapidly. The recorded current trace represents the pH change at the surface of the CBB, and the time constant of the pH-induced deactivation of the KcsA channel was fast (~ 30 ms)^31^. Closing of the last several channels are seen, lasting for 200 ms (Fig. 5A, inset). By switching the flow to the initial solution again (i.e., pH 4.0), the macroscopic current returned to the original steady-state level, indicating that the number of KcsA channels in the CBB was constant during the perfusion. These results show that the quick perfusion inside the bubble is sufficiently rapid to record the time course of the pH-dependent gating of the KcsA channel. Additionally, with a large pipette opening (30 μm in diameter) for the CBB, the electrode resistance is very low (~ 10 kΩ) compared with that of the patch-clamp (~ 1 MΩ), which reduces the background noise^33^. Accordingly, this CBB enabled simultaneous recordings of both macroscopic and single-channel currents in a single trace (Fig. 5A inset).

The wild-type (WT) KcsA channel exhibits the acid-induced activation followed by the inactivation such that the channel becomes gradually non-conductive even though the cytoplasmic side is kept acidic. Following the acidic jump, we successfully detected the inactivation process as an attenuation of the macroscopic current amplitude with a time course of seconds (Fig. 5B)^31^,^32^,^34^. In Fig. 5B, the time course of the channel current for a non-inactivating mutant, E71A, is superimposed. While E71A remained open as long as the cytoplasmic side was acidic, the activation time course was indistinguishable from that of the wild type.

## Conclusions

The CBB developed herein exploited the technical advantages of both the patch-clamp and DIB methods. Highly manipulable pipettes controlled by a motor drive allow fine positional adjustments of the bubbles, and pressure-control device generates bubbles of desired sizes. On the other hand, various types of bilayers with variable lipid composition were readily formed. In the CBB, bubbles as small as 50 μm in diameter were easily formed along with small bilayers, and the electrical background noise was dramatically decreased relative to that in earlier reports using DIB. The small size of the bubble also increases the perfusion speed, and repeated solution exchange is attained by the rapid perfusion-and-drainage system. Using this highly controllable system, we revealed the behaviour of the pTB channel in response to attach–detach transitions. The mechanical withdrawal of the membrane-embedded channels from the bilayer and the preferential distribution of the channels on one of the leaflets provided an idea how pTB is inserted into the membrane. The repeated reformability of the membrane in the CBB enabled this unprecedented experiment. The lipid bilayer techniques are briefly compared in supplemental Table S1.

Similar to manipulations of soap bubbles^1^, the CBB, with its ability to be physically manipulated, enables various geometrical configurations of the membrane. Changes in the intra-bubble pressure can deform the bilayer membrane into a convex and concave shape, and we are studying the effect of membrane curvature on channel activity.

## Methods

### Reagents

All chemicals, except for the phospholipids, were purchased from Nacalai Tesque (Kyoto, Japan). Azolectin (L-α-phosphatidylcholine type IV-S) was obtained from Sigma–Aldrich (St. Louis, MO, USA), and the other lipids (POPG and POPC) were from Avanti Polar Lipids (Alabaster, AL).

### Sample preparation

Purified polytheonamide B (pTB) was gifted from Dr. Matsunaga (Univ. Tokyo, Japan). pTB was solubilized in ethanol. Aliquots of the pTB solution were added to the electrolyte solution from which the bubbles were formed. The expression, purification and reconstitution into liposomes of the wild-type and mutant KcsA channels are described elsewhere^35^. Proteoliposomes were prepared by dilution as follows. First, liposomes were suspended in 200 mM KCl at a concentration of 2 mg/mL. Then, an aliquot of solubilized KcsA channel in 0.06% n-dodecyl-β-D-maltoside (DDM) was diluted 50 times by the liposome solution. The lipid/protein weight ratio of the proteoliposome was 2000 (for single-channel current measurements) or 20 (for macroscopic current measurements). The proteoliposome suspension was mixed with a small amount of concentrated buffer (pH 7.5 or 4.0) just before the experiments.

### CBB formation

All experiments were performed on an inverted microscope (IX71, Olympus, Tokyo, Japan), and pictures were recorded using a digital camera (Eos Kiss X5, Canon, Tokyo, Japan). Glass pipettes for the bubble formation and perfusion inside the bubble were fabricated from borosilicate capillary glass (OD/ID; 1.50/1.05 mm, Hilgenberg GmbH, Malsfeld, Germany) using a micropipette puller (P-87, Sutter Instrument, Novato, CA). For the bubble-forming pipette, the tip of the pipette was broken and slightly polished using a micro-forge (MF-830, Narishige, Tokyo, Japan). The pipettes were operated by motor-driven micromanipulators (MP-285, Sutter Instrument) under the microscope. The pressure in the pipettes was regulated by a high-speed pressure-clamp system (HSPC-1, ALA Scientific Instruments, Farmingdale, NY). Two methods for forming CBB were employed. One was the lipids-in-oil method (Fig. 1B) in which phospholipids to form CBB were supplemented from oil phase (20 mg/mL). The other one was the lipids-in-water method (Fig. 3A) in which phospholipids were supplemented from liposomes suspended in water phase (2 mg/mL). In both cases, two water bubbles were formed from glass pipettes by applying pressure inside the pipettes in the oil phase (150 μL) on the inverted microscope (Fig. 1A). The bubbles were kept separated for 2–3 minutes before contacting them to wait the lipid monolayer form on the water-oil interface. CBB was formed by contacting the bubbles and it was stable at least for our experimental period (2–3 hours) or under ± 400 mV membrane potential.

### Electrophysiologycal measurements

Electrical signals from ion channels in the CBB were measured as follows. The working electrode and the reference electrode were set inside two bubble-forming pipettes respectively. The membrane potential across the CBB was regulated and the ionic current was measured by using a patch-clamp amplifier (Axopatch 200B, Molecular Devices, Sunnyvale, CA). The current signals were filtered (2 kHz for the cutoff frequency) and sampled at 5 kHz by the amplifier. The sampled signals were digitized by an A/D converter (Digidata 1322A, Molecular Devices) and stored in PC by using software (pCLAMP, Molecular Devices).

### Fluorescence imaging during perfusion

For the fluorescence imaging of perfusion inside the bubble, one of the bubble-forming pipette was filled with water-soluble fluorescence probe (Qdot® 655 ITK™ carboxyl quantum dots, Thermo Fisher Scientific, Waltham, MA) and the fluorescence was observed by using WIG filter set (Olympus). After CBB formation, two injection pipettes (with or without fluorescence probe) were penetrated into the bubble filled with fluorescence solution. Perfusion was started by applying pressure to the injection pipette containing non-fluorescence solution and wash out of the fluorescence inside the bubble was recorded by a high-speed camera (FASTCAM-512PCI 32K, Photron, Tokyo, Japan). Time course of the fluorescence intensity at the vicinity of the CBB was quantified by an image analysis software (ImageJ). The experiment was repeated after refilling the fluorescence solution through the other injection pipette.

## Author Contributions

M.I. conducted experiments. M.I. and S.O. wrote the main manuscript text and prepared figures. All authors reviewed the manuscript.

## Supplementary Material

Supplementary Information Supplementary information
