# Supplementary material for: Contact Bubble Bilayers with Flush Drainage
Source: Sci Rep. 2015 Mar 16;5:9110. doi: 10.1038/srep09110 (PMC4360637; doi:10.1038/srep09110)
Supplement: Supplementary Information [file srep09110-s1.pdf]

# Supporting Information

## Contact Bubble Bilayers with Flush Drainage

Masayuki Iwamoto and Shigetoshi Oiki

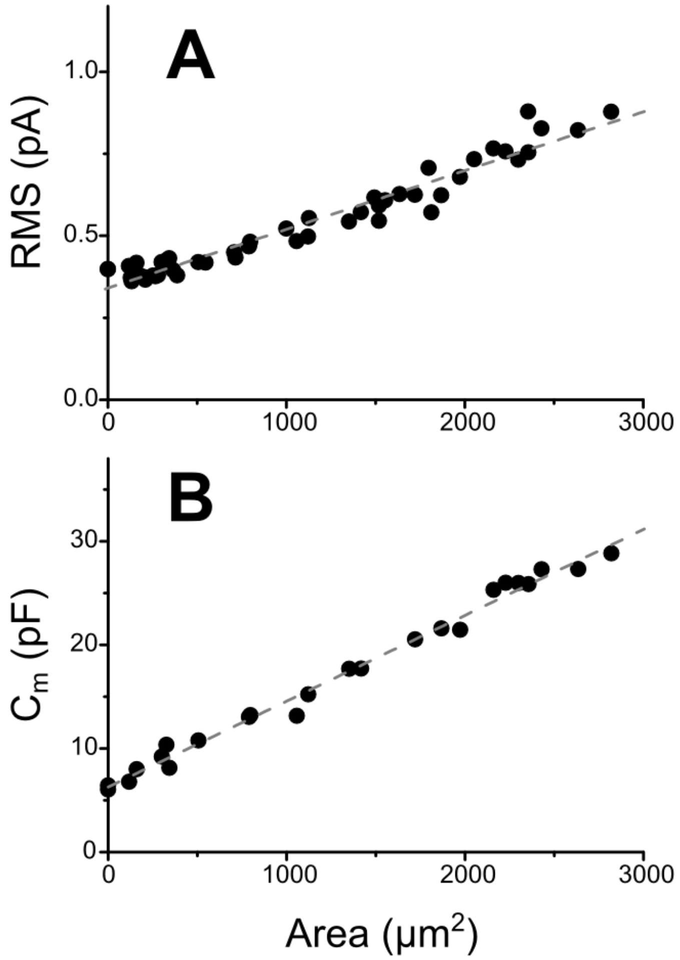


**Figure S1.** The rms noise (**A**) and the membrane capacitance (**B**) of the CBB as a function of the membrane area. Bubbles with the various size were formed in the azolectin-containing hexadecane and the diameter of the membrane was measured precisely under the microscope. (**A**) The rms noise was analyzed from the current trace under voltage-clamp condition where the membrane potential was held at 0 mV (cutoff filter, 2 kHz; sampling rate, 10 kHz). Almost liner relation was confirmed between the area of the CBB and the rms noise. The slope (1.8 x $10^{-4}$ pA/μm$^2$) indicates that the smaller membrane area of the CBB we usually prepare (10 μm diameter, ~80 μm$^2$) gains more than 1.4 pA reduction in the rms noise compared to conventional planar lipid bilayer (>100 μm diameter, > 8000 μm$^2$). (**B**) The membrane capacitance was estimated from a capacitive current obtained by the application of a ramp voltage command (±50 mV, 5 Hz). The slope (0.00829 pF/μm$^2$ or 0.829 μF/cm$^2$) represents the specific capacity and this value indicates that the formed bilayer is nearly solvent-free.

**Table S1. Characteristics of various lipid bilayer membranes**

| | Conventional PLB | Liposome patch | DIB | CBB |
|---|---|---|---|---|
| Immediate reformability[1] | yes | no | intermediate | yes |
| Asymmetric membrane | yes | no | yes | yes |
| Solution exchange[2] | slow | very fast | slow | fast |
| Background noise level[3] | high | low | high | low |
| Membrane deformation | no | no | no | yes[4] |
| Lipid applicability | diverse | limited[5] | diverse | diverse |

[1]Formation of a new membrane quickly after the breakdown of a membrane. Additionally, the membrane is formed repeatedly. [2]Approximate solution exchange times are <1 min for PLB, <1 ms for liposome patch, <5 sec for DIB and <20 ms for CBB. [3]The background noise level indicates the baseline current fluctuation (root means square, RMS) of 2 kHz cutoff filtered data. The RMS values are 0.67 pA for PLB, 0.32 pA for liposome patch, ~1.0 pA for DIB and 0.35 pA for CBB. For the value of DIB, we estimated from reported current trace in Syeda *et al.*, *J. Am. Chem. Soc.* 130 (2008) 15543-8. [4]A concave or convex membrane can be formed by applying a different inner pressure between the two bubbles. [5]Phospholipids that can form giant unilamellar vesicles can be used, but the giga-seal may not be attained.
